# Comparative study of thiolated and alkenylated functionalized β-CDs as highly mucopenetrating and mucoadhesive drug delivery systems

**DOI:** 10.1007/s13346-026-02063-3

**Published:** 2026-02-17

**Authors:** Effrosyni-Maria Kosti, Sophia D’Amico Huber, Alexander Mair, David Gintsburg, Andreas Bernkop-Schnürch, Gergely Kali

**Affiliations:** Center for Chemistry and Biomedicine, Department of Pharmaceutical Technology, Institute of Pharmacy, Leopold-Franzens-University of Innsbruck, Innrain 80/82, 6020 Innsbruck, Austria

**Keywords:** Mucoadhesion, Thiolated, Alkenylated, Oligosaccharides, Mucopenetration

## Abstract

Within this study, thiolated and alkenylated β-cyclodextrins were developed as novel mucoadhesive excipients capable of interacting with mucosal surfaces and prolonging the residence time of incorporated drugs at absorption sites, thereby potentially enhancing their bioavailability. For this purpose, cysteine-, cysteamine-, allylcarbamate-, and methacrylate-functionalized oligosaccharides were synthesized, and the resulting structures were identified by ^1^H NMR spectroscopy, Ellman’s and disulfide bond tests, and iodometry. The thiol and double bond content, as well as the stability of these functional groups at 37 °C, over 4 h, were evaluated. Alkenylated β-cyclodextrins showed significantly higher stability in aqueous solution compared to the thiolated products with oxidative sensitivity. Furthermore, the cytotoxicity and rheological, mucodiffusive, and mucoadhesive properties of the derivatives were assessed to elucidate their potential as multifunctional excipients for mucosal drug delivery. The cytotoxicity study confirmed that all derivatives were non-toxic within 4 h of incubation. Rheological measurements showed that β-CD highly modified with allylcarbamate exhibited a 15-fold increase in dynamic viscosity after incubating with intestinal mucus compared to the native β-CD. Regarding mucopenetration, β-CD cysteine was able to penetrate the mucus by more than 3% per 5 cm in 24 h. The results on porcine intestine revealed the superiority of thiolated derivatives in mucoadhesion, with at least 98% of the samples remaining on the intestinal tissue after 3 h of rinsing. For alkenylated β-CD with a similar degree of modification, low mucoadhesiveness was detected, but it was increased significantly with the degree of modification. The current study compares various mucoadhesive approaches and demonstrates that thiolated derivatives are more effective than alkenylated derivatives for mucosal drug delivery, but are less stable in physiological fluids.

**Graphical abstract:**

**Figure:**
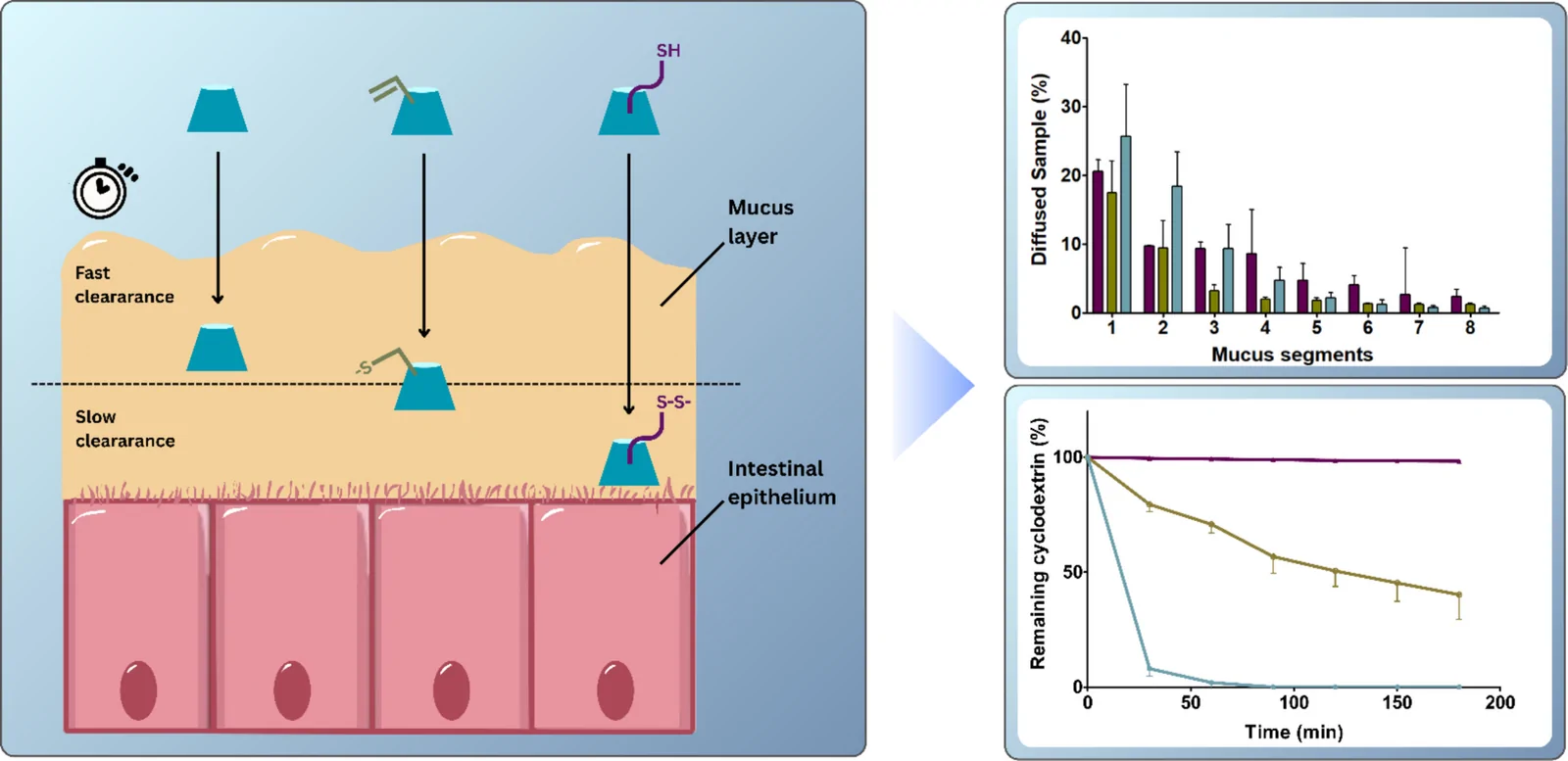

## Introduction

Mucosal drug delivery has gained considerable attention over the past decade as a promising alternative to the conventional parenteral administration route [1]. It offers several benefits, including improved patient compliance, avoidance of first-pass metabolism, and the ability to achieve both systemic and localized therapeutic effects [2–4]. Various mucosal sites—such as the nasal, buccal, ocular, vaginal, and gastrointestinal mucosa—provide accessible and physiologically favorable environments for drug absorption. Despite these benefits, a major challenge that limits the effectiveness of mucosal delivery systems is the relatively short residence time of formulations on the mucosal surface, which can lead to rapid clearance and reduced bioavailability [5, 6].

To overcome this limitation, extensive research has focused on strategies that enhance the interaction between drug carriers and the mucus layer. Among these, the development of mucoadhesive materials has proven particularly effective in prolonging retention and improving drug absorption [2, 5–12]. Mucoadhesion is primarily governed by physicochemical interactions between the delivery system and mucin glycoproteins in the mucus gel [13]. These interactions may involve both non-covalent forces—such as hydrogen bonding, van der Waals interactions, and electrostatic attraction—and covalent mechanisms, which can lead to stronger and more persistent adhesion [13]. Notably, covalent bonding, such as the formation of disulfide bonds between thiolated polymers and cysteine residues in mucin, typically results in a more stable and durable cross-linked network, thereby prolonging the residence time and overall performance of mucosal drug delivery systems [14].

Of particular interest are reactive oligo- and polysaccharide derivatives, which possess essential properties like aqueous solubility, biodegradability, and non-toxicity. The native oligosaccharide contains reactive functionalities, mainly hydroxyl groups, that can be derivatized with other functionalities, such as thiols, to enhance their mucoadhesiveness [15, 16]. Thiolated polysaccharides exhibit strong mucoadhesive properties because they react with cysteine-rich subdomains of mucus glycoproteins [17, 18]. In line with this, multiple studies have proven that thiolation of β-cyclodextrins (β-CDs) increases mucoadhesion, depending on the method and the degree of functionalization, up to 18-fold [19–22]. Additionally, macromolecules with double bonds are also evaluated as mucoadhesive excipients, because of the resulting thioether substructures in the reactions with cysteine moieties [17]. Several studies have shown that double bond moieties react with the thiol groups of mucus, thereby improving mucoadhesion, although to a lesser extent than thiolated oligomers and polymers [17, 23, 24]. It is noteworthy that there is no study comparing the various functional groups.

In this study, β-cyclodextrins (β-CDs) modified with various functional groups—cysteine, cysteamine, allyl, and methacrylate —were synthesized and comparatively evaluated. The derivatives were characterized in terms of degree of substitution, cytotoxicity, rheology, mucodiffusion, and mucoadhesive behavior. Building on previous findings from our group that showed polymers containing high densities of thiol and double bonds exhibit strong mucoadhesive properties [17, 25–27], this study aimed to highlight the differences in mucodiffusion and mucoadhesion of thiolated versus alkenylated β-CD derivatives in mucosal drug delivery applications.

## Materials and methods

### Materials

β-Cyclodextrin (β-CD) was purchased from Cyclolab, Hungary. Allylamine, dibutyltindilaurate (DBTDL), dimethylsulfoxide-*d*_*6*_ (DMSO-*d*_*6*_, 99.9 atom% D), cysteamine (Cya), L-cysteine (≥ 97%), methacryloyl chloride, triethylamine, pyridine (≥ 99.9%), and resazurin sodium salt were also provided by Sigma-Aldrich. Dimethylsulfoxide (DMSO, ≥ 99.5%) and Fluorescein isothiocyanate isomer I (FITC, ≥ 90% purity, HPLC) were used without further purification. Triton™ X-100, 5,5′-dithiobis (2-nitrobenzoic acid) (Ellman’s reagent), phenyl chloroformate (97%), minimum essential eagle medium (MEM), and acetone (suitable for HPLC, ≥ 99.9%) were obtained from Merck, Germany. ZelluTrans dialysis tube MWCO 1 kDa, 4-(2-hydroxyethyl)−1-piperazine ethanesulfonic acid (HEPES), and N,N-dimethylformamide (DMF, ≥ 99.5%) were obtained from Carl Roth, Austria. Sodium hydroxide (NaOH) was provided by Acros. Porcine small intestinal mucosa was kindly donated by a local slaughterhouse. FBS Supreme (South America origin, fetal bovine serum), Penicillin–Streptomycin (10.000 U/mL Penicillin, 10 mg/mL Streptomycin), and trypsin/EDTA (0.05%/0.02%) were purchased from Pan™ Biotech.

### Synthesis of cysteine, cysteamine, and allylcarbamate derivatives of β-CD

The β-CD derivatives were synthesized according to a method published by Lindemann and Heinze with minor modifications [15]. The synthesis is composed of two steps.

In the first step, a suspension of β-CD 1 g (0.88 mmol) in 10 mL of pyridine was stirred at room temperature until the cyclodextrin was finely dissolved. Then, phenyl chloroformate (3.87 mL, 30.84 mmol) was added dropwise over 1 h under magnetic stirring. After vigorous stirring overnight at room temperature, 200 mL of ice water was added to the reaction flask, and the precipitated modified β-CD was collected by filtration. The solid product was washed twice with 100 mL of cold demineralized water and once with 100 mL of ethanol. The phenylcarbonate-modified product was lyophilized at −80 °C under a pressure of 0.01 mbar for 48 h.

In the second step, 1 g of modified β-CD was dissolved in 13 mL of dry DMF (168 mmol). After complete dissolution, 2.10, 1.33, or 0.98 g of cysteine, cysteamine, or allylamine (17.23 mmol), respectively, was slowly syringed into the reaction mixture and stirred overnight at 60 °C under N_2_ atmosphere. The suspension was poured into 150 mL of cold acetone, and the derivative precipitated. The precipitate was then washed twice with 50 mL of cold acetone. The product was collected by filtration and freeze-dried at −80 °C under a pressure of 0.01 mbar for 48 h.

### Synthesis of methacrylate derivative of β-CD

β-CD was modified with methacryloyl chloride according to the previously outlined protocol [28] with minor modifications. The methacrylation of oligosaccharides was performed by dissolving 1 g of dry β-CD (0.88 mmol) in 8 mL of anhydrous dimethylformamide (DMF) under stirring. Then, 0.85 mL (6.10 mmol) of anhydrous triethylamine (TEA) was added. The mixture was stirred and cooled in an ice bath until it reached 0 °C. Next, a mixture of 500 μL of methacryloyl chloride and 0.38 mL of anhydrous DMF was added dropwise with intense stirring. The reaction was allowed to reach room temperature while stirring for 4 h gradually. After that, triethylamine hydrochloride was removed by filtration, and the filtrate was precipitated into cold acetone (80 mL) and centrifuged to obtain the solid product. The precipitate was washed with acetone three times (50 mL, each time) and filtered. Any trace of acetone was removed under vacuum, and the final product was freeze-dried at −80 °C under a pressure of 0.01 mbar for 48 h.

### Chemical characterizations

^1^H NMR measurements were done on a “Mars” 400 MHz Avance 4 Neo spectrometer from Bruker Corporation (Billerica, MA, USA, 400 MHz) in dimethyl sulfoxide‑d6 (DMSO‑d_6_) solution.

The thiol content of the target product was quantified using Ellman's and disulfide bond tests [26, 29]. For Ellman's test, 250 μL of Ellman's buffer (phosphate buffer 0.5 M, pH 8.0) and 500 μL of 5,5′-dithiobis (2-nitrobenzoic acid) solution (Ellman's reagent, 0.3 mg/mL in Ellman's buffer) were added to 1 mg of thiolated product and incubated for 2 h in the dark. Afterward, an aliquot of 100 μL was transferred to a 96-well plate, and absorption was measured at 450 nm using a microplate reader (Tecan infinite® M200 spectrophotometer, Tecan Austria GmbH, Grödig, Austria). The amount of free thiol groups was calculated from a calibration curve of L-cysteine under the same conditions. To quantify the amount of disulfide bonds, a disulfide bond test was performed. Thus, the procedure above was carried out after the reduction of disulfide bonds with aqueous 4% sodium borohydride (NaBH_4_) solution.

The double bond content of the alkenylated oligomers and polymers was determined by iodometry [17]. The β-CDs were first dissolved in chloroform and then reacted with iodine. After the addition of potassium iodide, the excessive amount of iodine was titrated with a 0.05 M sodium thiosulfate solution using starch as an indicator until the discoloration of the starch. The amount of double bonds was then determined using the formula provided below:

$$Iodine \;value \;of \;saccharide= \frac{(V_1-V_2) \times M \times 248.18}{w}\times 100 \;(g \;of\; iodine\; per\; 100 g \;of\; sample)$$

where w is the weight of the sample, V_1_ is the volume of the used titrant for analyzing the sample, V_2_ is the volume of the used titrant for analyzing the blank, and M is the molarity of Na_2_S_2_O_3_. The iodine value is used for the indirect determination of double bonds, since it quantifies the amount of iodine that is absorbed per 100 g of sample.

### Stability study of thiol groups and double bond content

The stability of the thiol and double bond content was evaluated by a method developed by our research group with minor modifications [30]. First, 10 mg of each modified β-CD was dissolved in 8 mL of 0.1 M phosphate buffer solution at pH 8. The solution was left shaking at 300 rpm and 37 °C for 4 h. Then, the number of free thiols and double bonds was quantified as previously described.

### Cytotoxicity studies on the Caco-2 cell line

Cytotoxicity screening was conducted using the resazurin assay as described in a previously published method [31]. Briefly, Caco-2 cells were seeded in a sterile 96-well plate at a density of 20,000 cells per well in minimum essential medium (MEM) supplemented with penicillin/streptomycin solution (100 units/0.1 mg/L) and 10% v/v fetal bovine serum (FBS). The cells were then incubated for 3 days at 37 °C under 5% CO_2_ and a 95% relative humidity environment in order to let them adhere to the bottom of the well.

The native and modified oligomers and polymer test solutions were prepared at concentrations of 0.05%, 0.1%, and 0.2% w/v in 25 mM HEPES buffered saline (HBS) at pH 7.4. The cells were washed twice with preheated HBS, then test solutions, along with pure HBS as the positive control and 1% v/v Triton™ X-100 as the negative control, were added in four replicates to the cell culture plate at volume of 100 μL per well and incubated at 37 °C in a 5% CO_2_ and 95% relative humidity environment for 4 and 24 h. After incubation, test solutions were removed, and cells were washed twice with preheated HBS pH 7.4. An aliquot of 150 μL of 2.2 mM resazurin solution was added to each well, and the cells were incubated again under the same conditions for 2 h. Afterward, 100 μL of supernatant from each well was transferred to a black well plate and measured at 540 nm of excitation wavelength and 590 nm of emission wavelength. The following equation calculates cell viability:

$$Cell \;viability \;\left(\%\right)=\frac{Experimental \;intensity-Negative\; control\; intensity}{Positive \;control \;intensity-Negative \;control \;intensity}\times 100\%$$

### Purification of small intestinal mucus

The pig's small intestine was cut into segments approximately 10 cm long and incised along its length. Parts containing chyme were removed. Mucus was gently scraped from the mucosal surface with a finger, collected, and stored on ice. In order to purify, the mucus was mixed with 0.1 M NaCl at a ratio of 1 g of mucus to 5 mL of NaCl, stirred gently on ice for 1 h, and centrifuged for 1 h at 4 °C at 10,400 g. Both the supernatant and pellet were discarded. The remaining mucus was resuspended in half the original volume of 0.1 M NaCl, stirred again on ice for 1 h, and centrifuged under the same conditions. After centrifugation, the purified mucus was ready for further use.

### Rheological investigations

Rheological characterization was performed using porcine intestinal mucosa to understand the interactions between the mucus and the samples by a plate-plate combination rheometer (Thermo Scientific™ HAAKE MARS™, Thermo Fisher Scientific, Karlsruhe, Germany; rotor PP35Ti, D = 35 mm, gap 0.5 mm) following a previously described method with minor modifications [20, 25]. The native and modified samples were measured at a concentration of 0.3% w/v in a 0.1 M phosphate buffer solution at pH 6.8. For a deeper understanding of the interaction sample-mucus, 100 µL of the sample solution was added to 0.5 g of freshly purified mucus, which was placed in a petri dish. Then, the petri dish was transferred to the incubator for 3 h at 37 °C to allow sufficient interaction between the mucus and the β-CDs. The gap between the cone and plate was adjusted to 0.049 mm, and the temperature was maintained at 37 °C. Oscillatory stress sweep tests were conducted within the linear viscoelastic range, applying shear stress between 0.01 and 50.0 Pa while maintaining a constant frequency of 1 Hz. These tests provided values for dynamic viscosity (ƞ), elastic modulus (G′), and viscous modulus (G″).

### Labeling with fluorescein isothiocyanate (FITC)

Native and modified β-CDs were fluorescently labeled with FITC. For this purpose, a labeling procedure adjusted from Knoll et al. was followed [32]. Briefly, 0.5 g of sample was dissolved in 100 mL of dimethyl sulfoxide (DMSO), and then 375 µL of dibutyltin dilaurate (DBTDL) and 6.25 mg of FITC were added, and the reaction mixture was stirred for 24 h at room temperature in the dark. Afterwards, the labeled β-CDs were transferred to regenerated cellulose dialysis bags (MWCO 1 kDa), and dialyzed until no fluorescence was observed in the dialysis water, indicating successful removal of free FITC. In the case of thiolated derivatives, the pH of the dialysis water was set to 6 with hydrochloric acid to prevent oxidation.

### Mucodiffusion studies

The diffusion properties of unmodified and modified oligomers and polymers into purified porcine intestinal mucus were evaluated using a rotating tube method, as described previously [33–35]. Briefly, 300 µL of purified porcine intestinal mucus was added to silicone tubes of 5 cm in length. FITC-labelled β-CD and its derivatives at a concentration of 1% m/v in 0.1 M phosphate buffer, pH 6.8, were prepared, and 50 µL of hydrated samples were added to the mucus-filled tubes. Both ends of the tubes were sealed with parafilm, and the tubes were rotated in an incubator at 37 °C for 24 h under light protection. Afterwards, the tubes were frozen at −80 °C and cut into 8 slices of 2 mm. To each slice, 350 µL of 0.1 M phosphate buffer at pH 6.8 was added and continuously shaken overnight. Then, the samples were centrifuged for 5 min at 13.400 rpm using a MiniSpin Centrifuge (Eppendorf, Hamburg, Germany). For control reasons, a 50 μL sample was added to 350 μL 0.1 M phosphate buffer, pH 6.8. The fluorescence intensities of 100 µL of the supernatant were measured at emission 530 nm and excitation at 490 nm. The experiment was conducted with five replicates.

### Ex vivo mucoadhesion study on porcine intestine

To investigate the adhesive properties of the prepared derivatives on the mucosal membrane, in vitro mucoadhesion studies were performed on porcine intestine (freshly collected from a local slaughterhouse) as previously described by our research group [29].

To evaluate mucoadhesive properties, the porcine intestine was cut into small pieces, approximately 3 × 2 cm, then it was opened lengthwise and fixed on a half-cut 50 mL Falcon tube using double-sided adhesive tape. The tubes were positioned at a 45° angle in an incubation chamber with 100% relative humidity. Afterward, the mucosa was continuously rinsed with 100 mM phosphate buffer at pH 6.8 for 15 min at a flow rate of 1 mL/min.

Then, 10 mg of the tested polymer was placed on the mucosal surface, and left for 10 min to react, after which the flow of phosphate buffer was resumed. The solution was collected at specific time points: 30, 60, 90, 120, 150, and 180 min. An aliquot of 1 mL from each solution was removed and centrifuged at 10.500 rpm for 10 min at room temperature (MiniSpin Centrifuge, Eppendorf, Hamburg, Germany). Finally, 0.1 mL of each sample was transferred to a black 96-well plate, and fluorescence intensity was measured at an emission wavelength of 490 nm and an excitation wavelength of 530 nm using a Spark® multifunctional microplate reader (Tecan Austria, GmbH). Later, 5 mg of FITC-labeled β-CDs and their derivatives were dissolved in 15 mL of this collected buffer, and this solution was used to calculate the percentage of remaining fluorescently labeled oligosaccharide on each mucosa.

All experiments were performed in triplicate. The results were calculated using the following equation:

$$Polymer \;remaining \;on\; the\; mucosa\; \left(\%\right)=\frac{Fluorescence\; sample}{Fluorescence\; control}\times 100$$

### Statistical analysis

All experiments were conducted in at least three replicates. Data analysis was carried out using GraphPad Prism 5 (GraphPad Software, Inc., San Diego, CA, USA). Results are presented as mean values ± standard deviation (SD). Statistical analysis was performed using one-way ANOVA and the two-way Bonferroni multiple comparisons test, with *p* < 0.05 considered statistically significant.

## Results and discussion

### Synthesis of cysteine, cysteamine, and allylcarbamate β-cyclodextrin (β-CD)

**Fig. 1:**
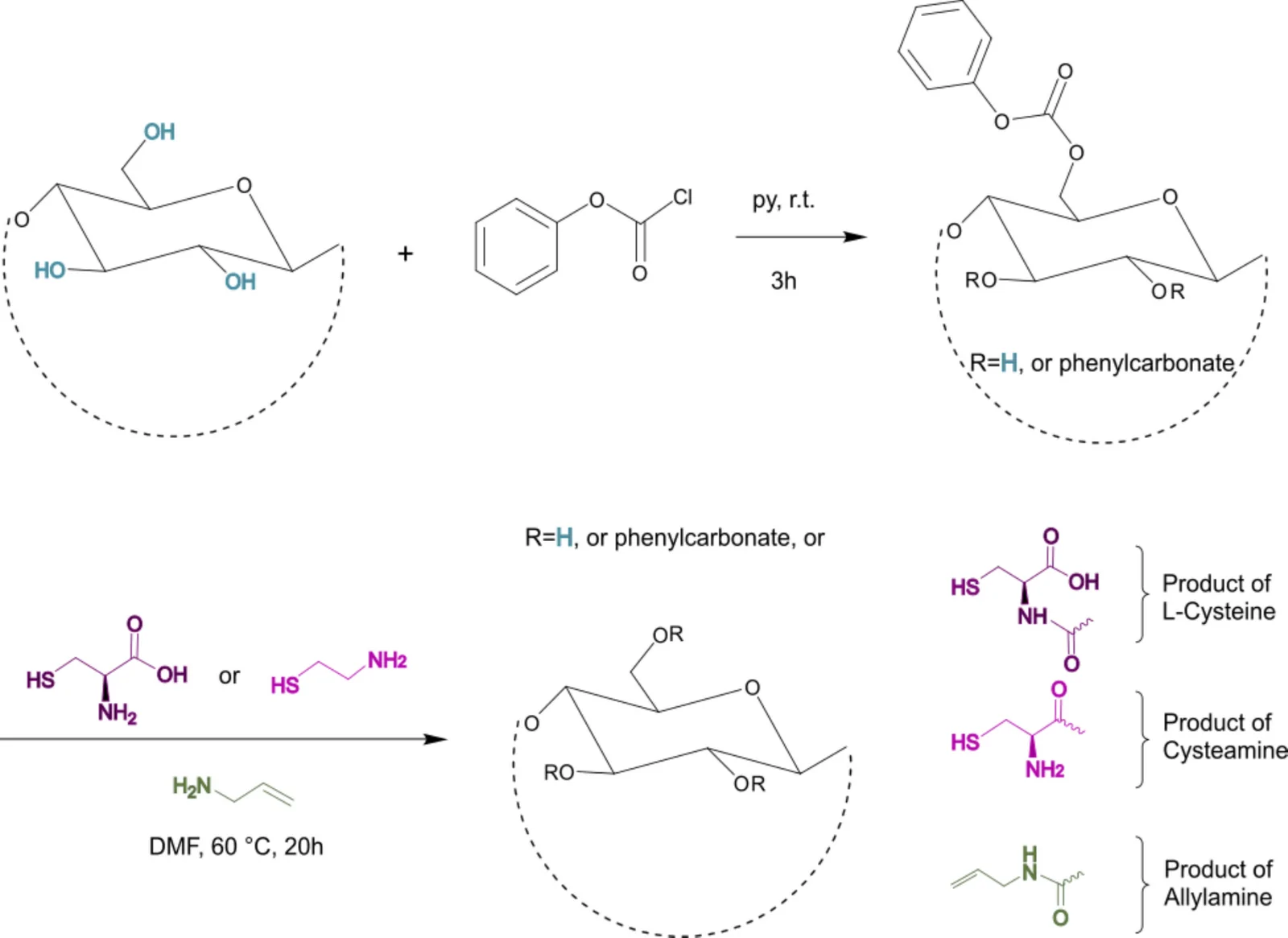
Schematic representation of the two-step synthesis of cysteine, cysteamine, and allylcarbamate-modified β-CDs. For clarity, the modification is shown on a single glucose unit of the β-CD structure

The most common routes for modifying oligosaccharides involve esterification or direct conversion of hydroxyl groups. The carbamate linkage is a rarely used modification for oligosaccharides, even though it could lead to a higher degree of modification that significantly affects the mucoadhesive properties. In this two-step synthesis, the hydroxyl groups of the repeating units were first reacted with phenyl chloroformate, which introduced a good leaving group. In the second step, the nucleophilic amine attacked the carbonyl of the phenyl carbonate derivative, resulting in the formation of the carbamate final product. The synthetic steps for the above modification are shown in Fig. 1.

### Synthesis of methacrylate β-cyclodextrin (β-CD)

**Fig. 2:**
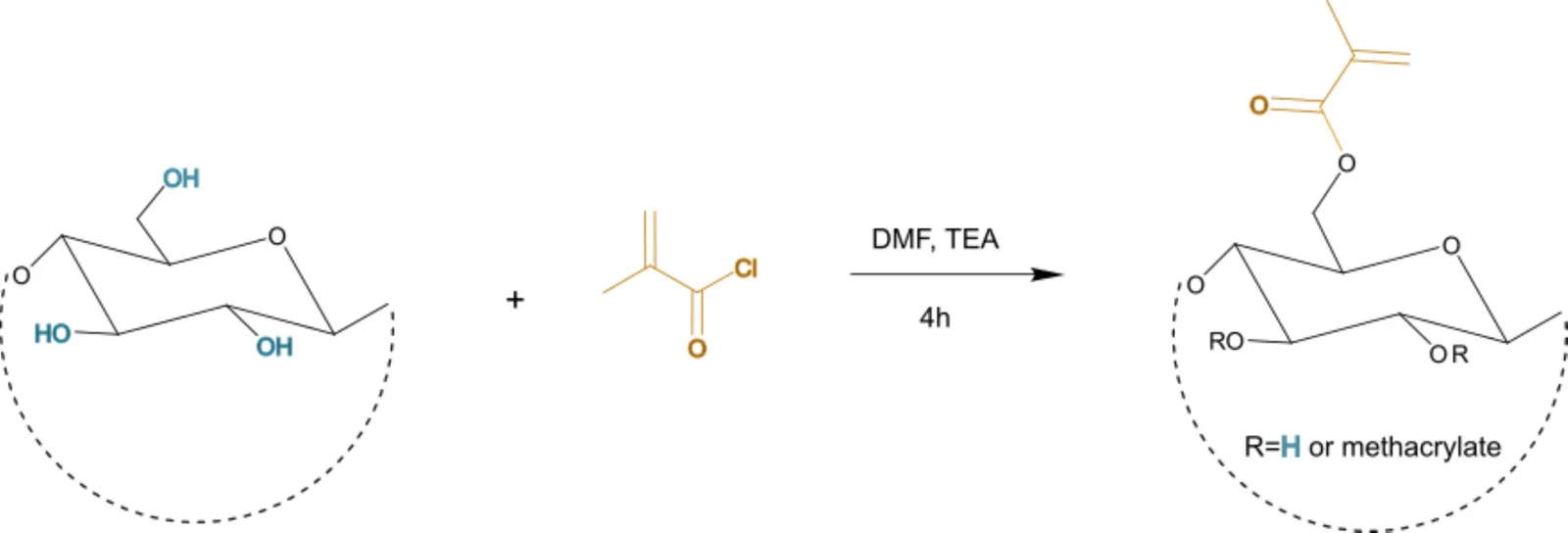
Schematic representation of the synthesis of methacrylate derivatives. For clarity, the modification is shown on a single glucose unit of the β-CD structure

Due to the low reactivity of the allylic moieties, methacrylates were also synthesized to screen the mucoadhesive and mucopenetrating properties of alkenylated oligosaccharides. To introduce reactive double bonds into the oligosaccharide structure, the native β-CD was reacted with methacryloyl chloride in the presence of DMF and TEA under magnetic stirring for 4 h. The chemical modification from the native to the synthesized β-CD is depicted in Fig. 2.

### Chemical characterization

The ^1^H NMR spectra of the derivatized β-CDs were recorded in DMSO-*d*_*6*_. β-CD was modified through a two-step reaction involving a phenylcarbonate intermediate. The ^1^H NMR spectrum of the intermediate confirmed the presence of aromatic protons at δ = 6.75–7.75 ppm, indicating a successful reaction with phenyl chloroformate. Moreover, successful conjugation of cysteine and cysteamine with the intermediate was confirmed by ^1^H NMR, showing methylene signals at δ = 2.5–3.0 ppm, consistent with the expected structures. In the case of cysteamine, splitting is also observed in the 2.5–3 ppm region, due to the presence of two magnetically non-equivalent, coupled methylene groups (*–CH₂*–C*H₂–*). Particularly, in the case of cysteine-modified β-CD, a very sharp peak appears at approximately 1.20 ppm, confirming the results from Ellman’s test regarding the high free thiol content.

Regarding allylcarbamate derivatives, the ^1^H NMR spectrum confirmed the presence of allylic protons in the range of δ = 5.6–5.9 ppm, slightly overlapping with the hydroxyl groups at C2 and C3. Furthermore, important peaks at 7.0–7.5 ppm are attributed to the *-NH-* proton of the carbamate, indicating that the allylcarbamate group was successfully attached.

In the case of the methacrylate derivative, the spectrum presents a peak at 1.85 ppm, indicating the presence of methyl protons of the methacrylate group. Additionally, the successful modification of β-CD methacrylate is indicated by the broad peaks observed between 5.70 and 6.20 ppm, corresponding to the double bond.

Ellman’s and disulfide tests were performed to quantify the amounts of free thiol groups and disulfide bonds of thiolated derivatives, respectively. The results are presented in Table 1.

**Table 1:** Quantification of the amount of total thiol/double bond content and the degree of substitution (DS) per β-CD

	Total thiol/double bond content (μmol/g)	Degree of Substitution (DS) per β-CD
*β-CD cysteine*	647.77 ± 47.95	0.74 ± 0.05
*β-CD cysteamine*	599.25 ± 147.82	0.68 ± 0.17
*β-CD allyl l-DS*	852.81 ± 43.12	0.97 ± 0.05
*β-CD allyl h-DS*	3937.14 ± 433.15	4.47 ± 0.49
*β-CD MAC*	2479.22 ± 170.72	2.81 ± 0.19

Having a high level of free thiol groups and a low amount of disulfide bonds is beneficial because, as established, an increase in the free thiol groups implies a better mucoadhesive profile [36, 37]. In both cases, a low amount, ≤ 10% of disulfide bonds, was detected. Regarding the difference in reactivity between the two thiolated ligands, cysteine appears to be incorporated slightly more effectively. The higher reactivity may be attributed to the carboxyl group, which influences the electronic environment of the amino group by stabilizing the deprotonated, nucleophilic amine species, allowing it to attack the electrophilic carbon of the phenyl carbonate derivative.

The amount of double bonds, which reflects the degree of substitution (DS), was determined by iodometric titration. It was observed that the β-CD alkenylated derivative had a similar, slightly higher DS compared to the thiolated ones. Because allylic compounds are expected to have lower mucoadhesive properties than thiolated CDs, β-CD allylcarbamate was resynthesized with a higher degree of substitution to promote stronger mucoadhesion. Methacrylate-functional β-CD was also synthesized for comparison, as this group forms more effective thioether bonds with cysteine-rich mucus glycoproteins than the allyl derivative. The degree of methacrylation was higher than the DS for the thiol derivatives and was close to that of the β-CD with high allyl content. Because of the presence of the carbonyl group and the comparatively high degree of substitution, β-CD MAC was readily soluble in aqueous solutions (10.00 ± 0.70 mg/mL).

### Stability study of thiol groups and double bond content

A stability study was carried out to compare whether products containing double bonds or thiol bonds, expected to show oxidative sensitivity, exhibit greater stability in aqueous solution. The experiment was conducted according to ICH Q1A (R2) guidelines for Stress Testing, at 37 °C, pH 8, and 300 rpm for 4 h [38]. This study was carried out to evaluate the physicochemical stability of modified β-CDs in physiological fluids and to quantify the amount of reactive moieties likely to be available in human intestinal fluids.

**Fig. 3:**
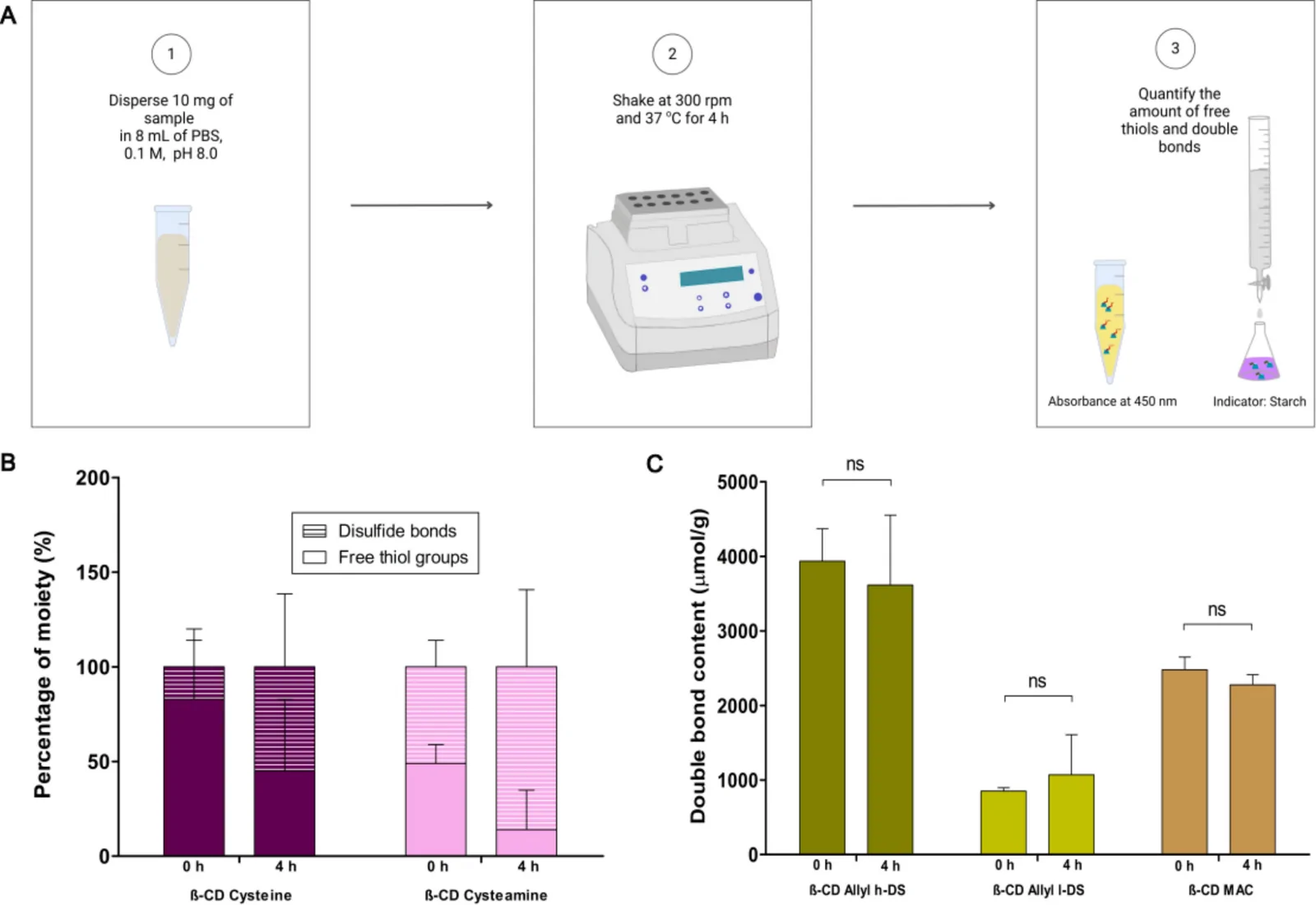
Stability of the **A**) Schematic illustration of the protocol for evaluating thiol groups and double bond content. **B**) Thiol groups of thiolated samples, and **C**) Double bonds of alkenylated samples, at pH 8. Measurements were conducted in a concentration of 1.25 mg/mL at 37 °C for 4 h. Indicated values are illustrated as means ± SD (*n* ≥ 3). Non-statistically significant differences are indicated with ns

In the case of thiolated samples, it was obvious that disulfide bonds were formed, and the number of free thiols was dramatically decreased (Fig. 3A). In contrast, the amount of double bonds of alkenylated β-CDs before and after 4 h of incubation in phosphate buffer was not statistically significantly different (Fig. 3B). This stability study highlights the significantly higher stability of alkenylated over thiolated β-CDs.

### Evaluation of cytotoxicity assay

The resazurin assay measures the amount of resazurin converted into resorufin, providing an indirect assessment of cell viability. As it refers to ISO 10993 about in vitro cytotoxicity testing, a cell viability above 80% is generally considered as non-cytotoxic [39].

**Fig. 4:**
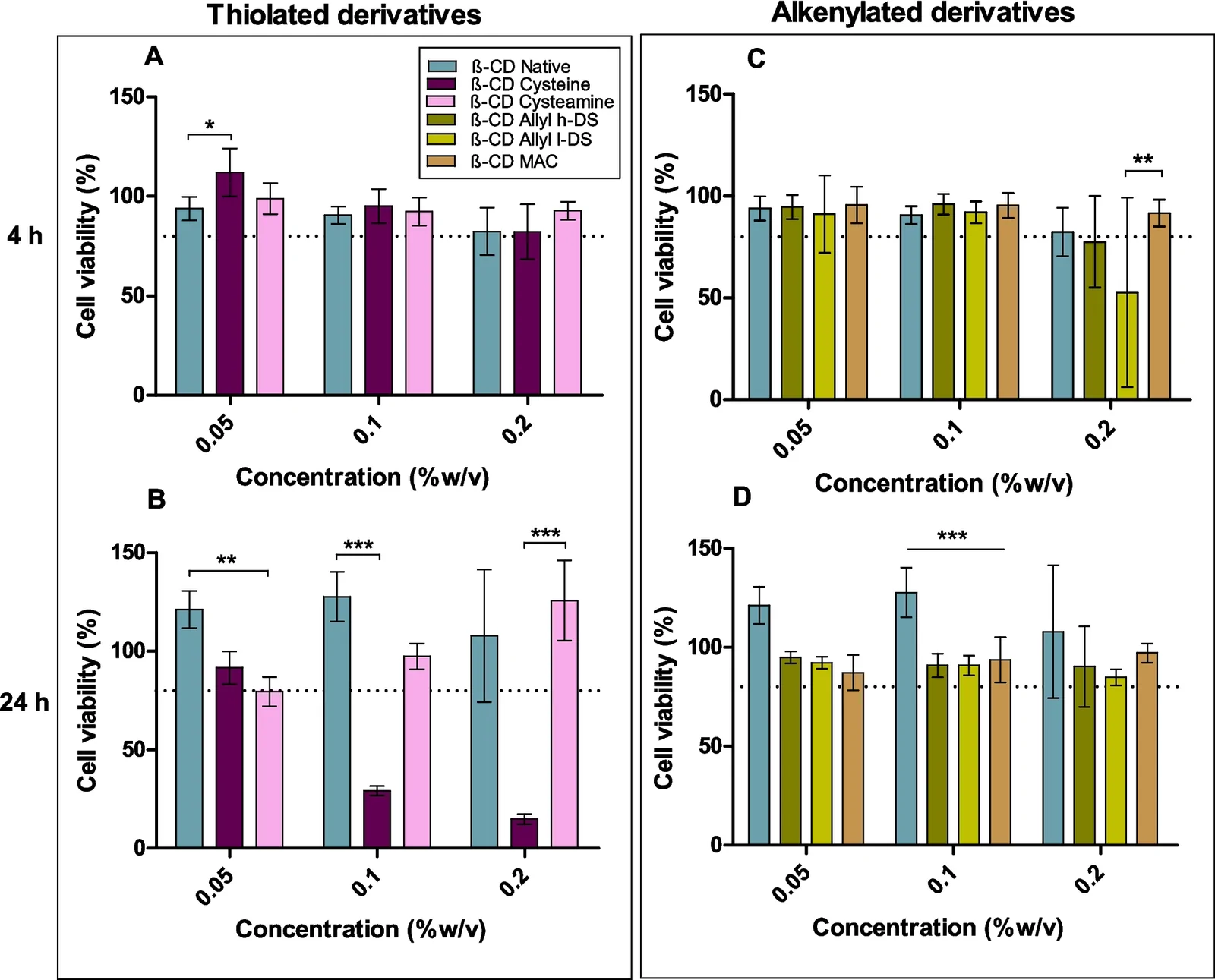
Cell viability of Caco-2 cells with thiolated and alkenylated derivatives after 4 h and 24 h incubation at 37 °C in an atmosphere of 5% CO_2_ and 95% relative humidity in different concentrations: 0.05, 0.1, 0.2% w/v. **A**) Cell viability of Caco-2 cells with native and thiolated derivatives: Native (light blue), cysteine (purple), and cysteamine (pink) functional β-CDs after 4 h incubation. **B**) Cell viability of Caco-2 cells with native and thiolated derivatives after 24 h incubation. **C**) Cell viability of Caco-2 cells with native and alkenylated derivatives: Native (light blue), allyl (green—low allyl content), allyl (olive green—high allyl content), and methacrylate (beige) functional β-CDs after 4 h incubation. **D**) Cell viability of Caco-2 cells with native and alkenylated derivatives after 24 h incubation. Indicated values are illustrated as means ± SD (*n* = 4). Significant differences are indicated as *p < 0.05, **p < 0.01, and ***p < 0.001

As displayed in Fig. 4, both thiolated and alkenylated derivatives appear to be safe for the cells after 4 h of incubation. Interestingly, β-CD cysteine exhibits a toxic profile after 24 h of incubation at concentrations of 0.1% and 0.2% w/v. Since β-CD native is tolerable by the cells (Fig. 4B), the high toxicity level of β-CD cysteine is likely due to its higher cellular uptake [40].

### Rheological investigation of samples with mucus

**Fig. 5:**
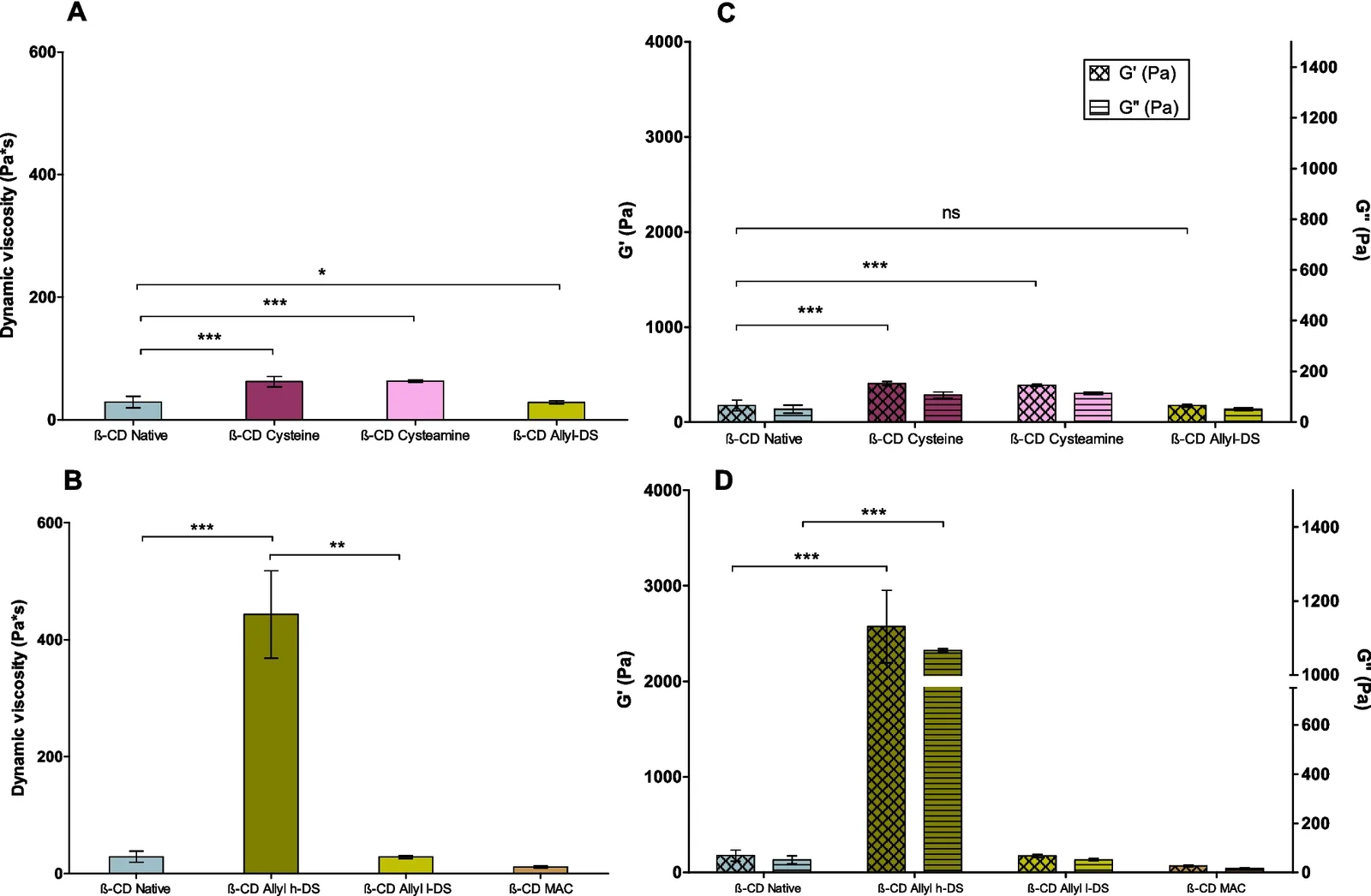
Rheological measurements of thiolated and alkenylated derivatives. **A**) Dynamic viscosity of native (light blue), cysteine (purple), cysteamine (pink), and allyl (green – low allyl content) functional β-CDs incubated with mucus in a weight ratio of 1:5 at 37 °C for 3 h. **B**) Dynamic viscosity of native (light blue), allyl (green—low allyl content), allyl (olive green—high allyl content), and methacrylate (beige) functional β-CDs incubated with mucus in a weight ratio of 1:5 at 37 °C for 3 h. **C**) Elastic modulus (G ‘) and viscous modulus (G’’) of low degree of substitution samples. **D**) Elastic modulus (G ‘) and viscous modulus (G’’) of high degree of substitution samples. Samples were prepared in 0.1 M phosphate buffer, pH 6.8. Indicated values are means ± standard deviation of at least three experiments. Significant differences are indicated as * *p* < 0.05, ** *p* < 0.01, and *** *p* < 0.001, while non-statistical significant differences with ns

The rheological measurement was performed with a cone-plate measuring system, and dynamic viscosity, storage modulus (G’), and loss modulus (G”) were recorded. Storage modulus (G’) refers to the sample stiffness, while loss modulus (G”) relates to its “liquid” properties [41]. These rheological properties provide an initial estimate of the interaction between the sample and the mucus, helping to explain its potential mucoadhesive capacity. Notably, both native and modified samples have G’ > G”, indicating that they are viscoelastic solids and modification did not significantly alter the elastic properties of the mixture (Fig. 5). Moreover, in the cases of both thiolated derivatives, the dynamic viscosity increased by 1.6–2.1-fold compared to native oligosaccharides, suggesting that disulfide bonds are formed with the substrate. The allylcarbonate functional β-CD (β-CD allyl l-DS) exhibited lower mucoadhesive potential compared to thiolated oligosaccharides due to the low reactivity of the allylic group toward the cysteines in mucus. Therefore, β-CD with a higher DS was synthesized (β-CD allyl h-DS) to investigate the effect of the DS on mucoadhesive properties. The formed β-CD allyl h-DS showed a statistically significant increase in dynamic viscosity due to the higher degree of modification, reaching up to 15-fold enhancement in viscosity and 15-, 21-fold increase of G’ and G’’, respectively, compared to native β-CD. This reveals a strong interaction between the β-CD allyl h-DS and the mucus. Due to the relatively high reactivity of methacrylates (MAC) with thiols, β-CD was also modified with this functional group.

In the case of this latter β-CD, the dynamic viscosity was found to be quite low. Previous studies have shown that methacrylate groups can decrease the viscosity of polymethylmethacrylate systems by reducing the entanglements that are likely to form [42]. Even though MAC is a more reactive moiety, and β-CD MAC had a similar degree of substitution to β-CD allyl h-DS, their dynamic viscosities and subsequently their interaction with mucus were statistically significantly different. The effects of these viscosity differences on mucoadhesive and mucodiffusive properties will be further discussed in the next paragraphs.

### In vitro permeation studies with porcine intestinal mucosa

Diffusion, i.e., the penetrating mechanism, where the native and modified β-CDs move along the concentration gradient in the mucus [43], was examined using an experiment design called the “*rotating tube method’’*. Fluorescently labeled native and functionalized β-CDs were subjected to this study, and their amounts at various depths in the mucus layer were determined.

**Fig. 6:**
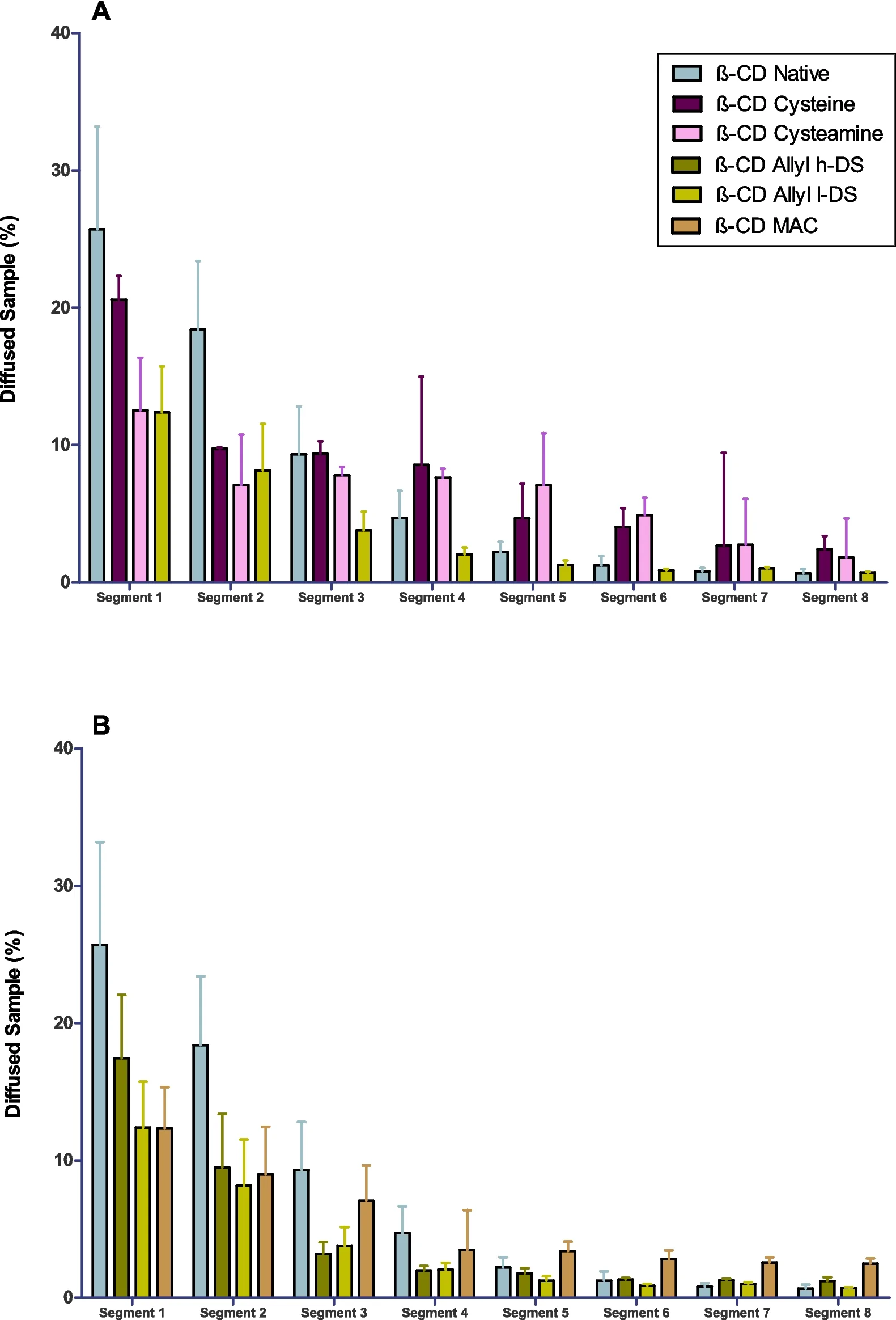
Percentage of diffused samples. **A**) Percentage of native (light blue), cysteine (purple), cysteamine (pink), and allyl (green—low allyl content) functional β-CDs across mucus after 24 h. **B**) Percentage of native and native (light blue), allyl (green—low allyl content), allyl (olive green- high allyl content), and methacrylate (beige) functional β-CDs across mucus after 24 h. Indicated values are means ± standard deviation of five experiments

The native β-CD diffused freely in the first segments (Fig. 6), as it was expected based on previous results [44]. Even though the rheological studies of the thiolated β-CDs indicated no statistically significant differences between them, β-CD cysteine exhibited the best mucopenetrating ability, reaching 2.42 ± 0.96% of the applied derivative in the last segment compared to only 1.82 ± 2.85% of the cysteamine-modified β-CD (Fig. 6A). This small difference is likely due to the presence of a free carboxyl group, resulting in a slightly negative character for this β-CD derivative. This led to reduced interaction with the negatively charged mucus via electrostatic repulsion, thereby enhancing its mucodiffusion properties. The favorable mucodiffusion profile of β-CD cysteine aligns with its strong mucoadhesive properties.

For the alkenylated β-CDs, allyl functional β-CDs possessed limited mobility through the mucus, particularly beyond the third segment (Fig. 6B). For β-CD allyl h-DS, this limited mobility may be attributed to its high dynamic viscosity that restricts its movement through the mucus. Notably, the β-CD MAC sample showed the highest concentrations, 2.50 ± 0.39% towards the latter part of the simulated mucus barrier. This can be explained by its low dynamic viscosity, which enables better mucopenetration in the mucus, and correlates with its prolonged mucosal residence time.

### Ex vivo mucoadhesion study

Numerous studies in the literature deal with the mucoadhesive properties of thiolated cyclodextrins [21, 25, 32] as well as alkenylated polymers [17, 45–47].

It has already been described that thiolated oligosaccharides can form disulfide bonds with cysteine-rich regions of mucin, while alkenyls form thioether bonds with them [36, 48]. Mucoadhesion studies were conducted to verify the potential application of these newly synthesized thiolated and alkenylated oligosaccharides.

**Fig. 7:**
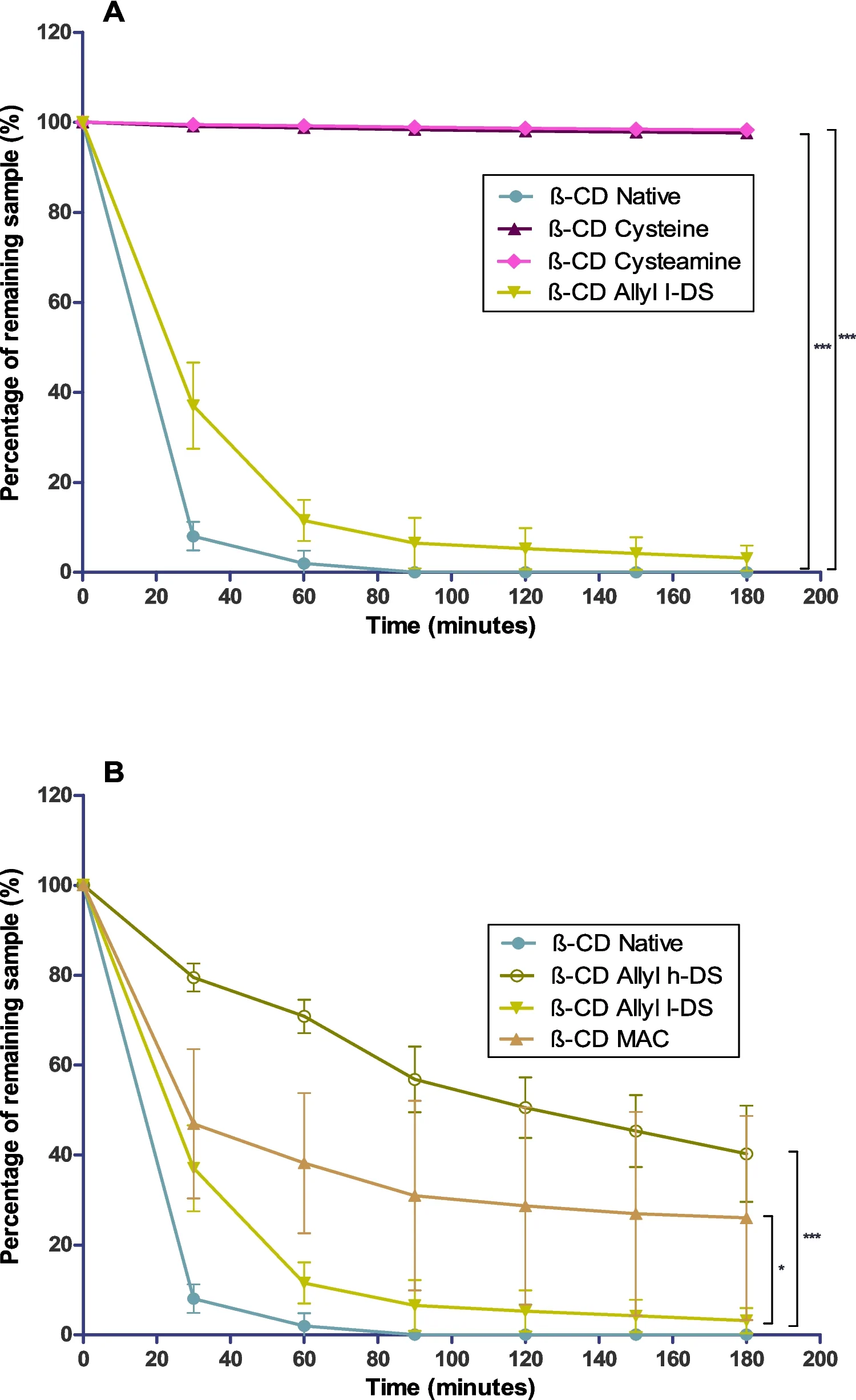
Schematic representation of the measurement of ex vivo mucoadhesion of **A**) Native (light blue circle), cysteine (purple triangle-up), cysteamine (pink rhombus), and allyl (green – low allyl content triangle-down), and **B**) Native (light blue circle), allyl (green—low allyl content triangle-down), allyl (olive green—high allyl content circle), and methacrylate (beige triangle-up) functional β-CDs. Significant differences are indicated as ∗ *p* < 0.05 and *** *p* < 0.001

Thiolated polymers easily undergo simple oxidation to form disulfide bonds with cysteine moieties in mucus, while the reactivity of allyl functionalities toward these cysteines was comparatively low. Therefore, the thiolated system was expected to perform more effectively. As depicted in Fig. 7A, thiolated β-CDs indeed exhibited high mucoadhesive potential and remained longer on the intestinal mucosa tissue. Compared to native β-CD, which was completely eliminated within 60 min from the intestine, both thiolated β-CDs were retained at above 97% on the mucosal surface after 180 min of rinsing. In contrast, β-CD allyl l-DS displayed a comparatively lower mucoadhesion and was rinsed off entirely within 3 h.

Regarding alkenylated β-CDs, the degree of substitution appears to be crucial for mucoadhesiveness. Accordingly, β-CD allyl h-DS remained on the mucosa longer than β-CD MAC, while β-CD allyl l-DS showed the shortest residence time (Fig. 7B). For β-CD allyl h-DS, this result is reasonable due to its high dynamic viscosity in mucus. Although the dynamic viscosity of β-CD methacrylate (β-CD MAC) was relatively low, fewer entanglements likely form due to stereochemical reasons. Mucoadhesion depends not only on rheological properties but also on the diffusion ability and reactivity of functional groups. Mucoadhesive carriers that penetrate deeper into mucus and form covalent bonds there, rather than merely attaching to the loose outer mucus layers, are expected to perform better and have prolonged residence time. Therefore, we hypothesized that the strong mucoadhesive property of β-CD MAC was due to its ability to diffuse significantly in the intestinal mucosa.

Compared to alkenylated β-CDs, the thiolated ones demonstrated a higher mucoadhesive ability. Although, as has been published multiple times in recent years, macromolecules with high double bond content can also be mucoadhesive [17, 49, 50], and the thiol-ene reaction involving the thiol groups of cysteine-rich regions in mucus glycoproteins and the alkenylated substances leads to the formation of a thioether linkage [17]. Our results align with a recent study [17], which examines the differences between a thiolated and an unsaturated polymer. However, even in this case, the double bond content is significantly higher than the thiol content; nonetheless, thiolated oligosaccharides remained longer on the mucosa.

## Conclusion

In this comparative study, the mucoadhesive properties and mucodiffusion of different thiolated and alkenylated cyclodextrins were systematically evaluated to identify the most effective functionalities for an oral drug delivery system. In particular, derivatives of β-CD with cysteine, cysteamine, allyl, and methacrylate were synthesized, and their structure and functional group content were confirmed via ^1^H NMR spectroscopy, and Ellman’s, as well as Iodine tests, respectively. Stability study revealed that alkenylated β-CDs exhibit greater stability during storage compared to thiolated ones. All the products exhibited low cytotoxicity within 4 h of incubation across a wide range of concentrations. Rheological studies confirmed the viscoelastic nature of the mucus sample mixtures and showed that the dynamic viscosity was slightly higher compared to the native β-CD, indicating their interaction with the intestinal mucosa. Regarding mucopenetrating properties, cysteine and methacrylate derivatives of β-CD demonstrated higher ability to diffuse through the mucus than those of allyl or cysteamine. This feature may be advantageous when thiolated β-CDs are employed as excipients, as they can enhance drug absorption. The ex vivo mucoadhesion study demonstrated that thiolated β-CDs exhibit a high mucoadhesive profile, due to their prolonged residence time on mucosal tissue, with a maximum loss of 2% over 3 h. In contrast, for alkenylated β-CD, this prolonged residence time was not achieved, but mucoadhesion increased with the degree of substitution.

This comparative study demonstrated various beneficial properties of thiolated and alkenylated β-CDs. Thiolated oligomers demonstrate the longest residence time on intestinal mucosa, while alkenylated β-CDs exhibit high stability in aqueous solutions. Despite these differences, both types of functional β-CDs hold great potential for oral drug delivery.

## Data Availability

Data available on request to the corresponding author.
